# Automated Mouse Pupil Size Measurement System to Assess Locus Coeruleus Activity with a Deep Learning-Based Approach

**DOI:** 10.3390/s21217106

**Published:** 2021-10-26

**Authors:** Alejandro Lara-Doña, Sonia Torres-Sanchez, Blanca Priego-Torres, Esther Berrocoso, Daniel Sanchez-Morillo

**Affiliations:** 1 Biomedical Engineering and Telemedicine Research Group, Systems and Automation Engineering Area, Department of Automation Engineering, Electronics and Computer Architecture and Networks, Universidad de Cádiz, 11009 Cádiz, Spain; alejandro.lara@uca.es (A.L.-D.); blanca.priego@uca.es (B.P.-T.); 2 Instituto de Investigación e Innovación Biomédica de Cádiz (INiBICA), 11009 Cádiz, Spain; sonia.torres@uca.es (S.T.-S.); esther.berrocoso@uca.es (E.B.); 3 Neuropsychopharmacology & Psychobiology Research Group, Psychobiology Area, Department of Psychology, Universidad de Cádiz, 11003 Cádiz, Spain; 4 Centro de Investigación Biomédica en Red de Salud Mental (CIBERSAM), Instituto de Salud Carlos III, 28029 Madrid, Spain

**Keywords:** pupillometry, locus coeruleus, pupil size, image processing, deep learning, machine learning

## Abstract

Strong evidence from studies on primates and rodents shows that changes in pupil diameter may reflect neural activity in the locus coeruleus (LC). Pupillometry is the only available non-invasive technique that could be used as a reliable and easily accessible real-time biomarker of changes in the in vivo activity of the LC. However, the application of pupillometry to preclinical research in rodents is not yet fully standardized. A lack of consensus on the technical specifications of some of the components used for image recording or positioning of the animal and cameras have been recorded in recent scientific literature. In this study, a novel pupillometry system to indirectly assess, in real-time, the function of the LC in anesthetized rodents is presented. The system comprises a deep learning SOLOv2 instance-based fast segmentation framework and a platform designed to place the experimental subject, the video cameras for data acquisition, and the light source. The performance of the proposed setup was assessed and compared to other baseline methods using a validation and an external test set. In the latter, the calculated intersection over the union was 0.93 and the mean absolute percentage error was 1.89% for the selected method. The Bland–Altman analysis depicted an excellent agreement. The results confirmed a high accuracy that makes the system suitable for real-time pupil size tracking, regardless of the pupil’s size, light intensity, or any features typical of the recording process in sedated mice. The framework could be used in any neurophysiological study with sedated or fixed-head animals.

## 1. Introduction

Strong evidence from studies in humans, primates, and rodents, indicates that variations in pupil size—not induced by changes in illumination—are correlated with arousal, attention, and cognitive processing [1,2,3,4,5]. More specifically, recent evidence in primates and rodents has shown that changes in pupil diameter can reflect neural activity of the locus coeruleus (LC) [6,7,8,9].

The noradrenergic nucleus LC is a brainstem structure, being the main source of norepinephrine/noradrenaline that participates in the modulation of arousal, attention, memory formation, stress response, or pain among other brain processes [10,11,12,13]. Additionally, changes in LC functionality has been associated with several neurodegenerative and neuropsychiatric disorders, such as Alzheimer’s disease, Parkinson’s disease, and chronic pain [12,14,15,16,17]. Particularly, in the pain field, LC involvement in the mechanisms underlying the comorbidity between chronic pain and mood disorders have been shown in animal models, stimulating the need to study the role of the LC as a critical center for chronic pain-induced anxiodepressive disorders [12,18,19]. Regarding neurodegenerative pathologies, LC is among the first brain structures to suffer degeneration. Thus, LC activity assessment is of huge interest as a potential early biomarker of neurodegenerative diseases, including Alzheimer’s and Parkinson’s diseases [15,20].

In this context, the study of LC using rodent models is a topic of current interest under investigation. Nevertheless, the difficulty of recording electrophysiological signals from a such small nucleus (1500 neurons per hemisphere in rodents) located deep in the pontine brain region makes conducting studies a challenge, both in animals and humans [11,21,22]. In this sense, pupil diameter monitoring could be a suitable assessment of LC function, since LC activity changes measured by fMRI correlates with pupil size fluctuations [9]. In fact, the cause–effect relationship between LC activation and pupil size has been recently evidenced by findings that stimulation of the LC, by electrical impulses, optogenetic techniques, or by designer receptors exclusively activated by designer drug (DREADD)-based chemogenetic tools, causes pupil dilation [6,7,21,23,24]. In addition, noxious stimulus was demonstrated to evoke pupil dilation, along with LC activation [24], suggesting a possible clinical utility for patients suffering pain. Nevertheless, it should be noted that pupil size is not only affected by noradrenergic, but also by other neurotransmitter systems, such as the cholinergic system [6], and changes in pupil size should be interpreted cautiously.

Therefore, pupillometry is the only available non-invasive technique that could be used as a reliable and easily accessible real-time biomarker of changes in the in vivo activity of the LC-noradrenergic system, with great potential for clinical translationality. As such, several studies have evaluated pupil fluctuations as a reflex of an LC-noradrenergic system activity, to study its involvement in cognitive tasks [25], memory performance [26], Alzheimer’s disease [27], and post-traumatic stress disorder [28]. Nevertheless, while it is common to find studies using pupillometry as a technique for the assessment of LC activity in humans, the extension of this technique to preclinical research in rodents is not yet widespread [21], despite the evidence that pupillometry can provide a useful in vivo assessment of LC function in awake or anesthetized rodents [29,30].

Moreover, in recent scientific literature, there is a lack of consensus on the technical specifications of some components used for image recording or positioning of the animal and cameras, with studies using different vision technologies, image sizes, frame rates, and light sources [6,21,23,24,30,31,32]. Recently, in attempts to propose common protocols, procedures describing how to perform pupillometry recordings in darkness, to assess modulation of the LC-NA system in awake and anesthetized mice, have been presented [21].

In this study, we present and assess a novel, robust, and reliable pupillometry system and a deep learning (DL) pipeline that can be used to indirectly assess, in real-time, the function of the LC-noradrenergic system in anesthetized rodents. The aim is three-fold. First, we present a novel hardware platform to perform the laboratory experiments, which allows positioning the animal, driving anesthesia, and adjusting the cameras for the best binocular pupils recording. Second, we describe a software tool to acquire bilateral recordings, optimizing parameters to minimize dropped frames and image quality. Finally, we propose a DL instance-based fast segmentation framework to calculate pupil diameter in mice in low-light settings. The performance of the proposed setup is assessed and compared to other baseline methods using a validation and an external test set. The goal is to provide an integrated and validated framework to enabling the non-invasive automatic study of the correlation of the pupil size and LC activity in lightly anesthetized rodents.

## 2. Recent Related Work

Pupil size is the result of the balanced action of two iris muscles: the sphincter and the dilator. These muscles are innervated by sympathetic neurons from the superior cervical ganglion and by parasympathetic neurons from the ciliary ganglion [33].

In rodents, pupillometry is conventionally applied as a non-invasive technique, with a non-fully automated assessment of pupil size changes during the experiment. This analysis is usually performed offline, frame-by-frame, making it time-consuming, labor-intensive, and inefficient in the case of long recordings.

The application of digital image processing techniques has attempted to alleviate this burden by transforming the annotation process into an automatic process. Conventional algorithms have been used in recent years to segment the pupil into circular or elliptical regions of interest. Table 1 summarizes the characteristics of some relevant studies carried out in rodents in the last five years linking pupillometry and LC activity. In 2016, Reimer et al. [6] explored the relationship among exploratory behaviors, cortical synchronization, and pupil dilation in adult mice. Later, in 2017, Liu et al. [24] explored how sympathetic and parasympathetic pathways contribute to pupil dilation induced by LC activation in adult rats using a pupillometry system assembled in-house. Yüzgeç et al. [32] studied, in 2018, the pupil size, coupling to cortical states and stability of deep-sleep in adult mice. In 2019, Breton-Provencher and Sur [30] analyzed mice mechanisms that related LC-noradrenergic activity and pupil dilation in awake head-restrained mice. Zerbi et al. [23], also in 2019, studied the effect in adult mice of LC stimulation on large-scale functional connectivity, assessing successful LC activation using pupillometry. In 2020, Hayat et al. [31] studied if optogenetic LC excitation elicits behavioral, electrophysiological, and pupillary signs of arousal in adult rats. Finally, and also in 2020, Privitera et al. [21] designed and implemented a complete pupillometry toolbox for real-time monitoring of LC activity in rodent. Their approach was two-fold. First, they described an image analysis pipeline using MATLAB. As an alternative and novel approach, the authors also proposed an adaptation of the motion-tracking software DeepLabCut (DLC), which included a deep neural network [34] for pupil segmentation and tracking.

Frequent features in most of the above-mentioned studies include the use of a conventional image analysis pipeline, as well as the need for manual adjustment of some parameters in some image processing stages, especially those related to thresholding, whisking, and noise. The unavailability of a systematic assessment to provide the degree of certainty on the performance of the pupil size detection algorithms was also a constant in the published studies. Only Breton-Provencher and Sur [30] provided a qualitative assessment of the results.

In addition to the need for manual adjustment of some parameters, the performance of algorithms based on conventional image processing techniques is conditioned by the low-light environment, common in these experimental settings. The limitations of such approaches are accentuated by mice vibrissae, blinking, and by opacities caused by the gel applied over the experimental subjects’ eyes to avoid corneal damage during light anesthesia. An approach that overcomes these drawbacks and minimizes or eliminates manual intervention is therefore required to reliably conduct experiments with rodents. In this regard, the application of DL was proven to be an effective approach for real-time pupil detection in humans [35,36,37,38,39]. These human studies are overwhelmingly oriented toward estimating pupil centers to determine gaze. Furthermore, pupillometry in rodents presents some challenges different from those encountered in humans. In rodents, the accuracy in defining the pupil size is affected by scattering movements, low contrast difference between the pupil and iris, blur, reflections, and noise [40].

Very recently, DL was applied in mice in LC studies [21]. However, although in their work, the authors provide access to a pre-trained DLC network, their study does not include the assessment of the algorithm accuracy for binding the average estimation error made in calculating pupil size. While the average tracking performance of DLC, an approach suited to track and locate arbitrary objects on an image, was evaluated in [34], this evaluation focused on the ability of the method to detect the body parts of interest for odor guided navigation and in demonstrating that deep architecture may achieve good performance.

## 3. Materials and Methods

In accordance with the objectives stated in the introduction, the materials and methods used in this study are described in this section.

### 3.1. Mechanical Framework and Hardware

Monitoring and evaluating changes in pupil dilation is necessary to keep the animal in a certain position within the viewing area of the recording cameras. Different animal fixation devices are used according to experimental needs. Existing systems are scarce and of high cost. Non-invasive techniques, such as cranial fixation by means of stereotaxic platforms [21,31] are commonly used. However, these devices are often not designed to accommodate all of the necessary elements (cameras, lenses, connecting cables, etc.). In the existing literature, there is no consensus on the arrangement of these elements, their shape, or characteristics, which is why we prioritized parameters, such as the platform flexibility, to have the greatest degree of freedom to comfortably arrange the equipment. This flexibility facilitates the definition and subsequent refinement of the method for acquiring experimental data.

Based on the functional specifications emanating from the research needs, a system was designed to place the experimental subject, the video cameras for data acquisition, and a light source. This system consists of a steel base plate in which were placed: (a) a height-adjustable platform to place the lightly anesthetized rodent; (b) two cameras to record the rodent’s eyes; and (c) a structure that allows the cameras to be positioned at any spatial coordinate within the platform volume. The base support in which the rodent is placed was designed specifically for the intended use. It was manufactured by 3D printing in polyethylene terephthalate glycol (PETG). PETG was selected because of its biocompatibility, considering that it can be sterilized after each test using disinfectants commonly available in animal experimentation laboratories, such as acetone or isopropyl alcohol.

A CMOS camera (DCC1545M, Thorlabs, Germany) was selected for image recording. It was a 1.3 megapixel monochrome camera that was used in recent studies [30]. This camera is capable of capturing images at a rate of up to 25 fps, with a resolution of 1280 × 1024 pixels. A lens of 25.0 mm focal length (Edmund Optics, UK), without infrared filter and with an aperture of f/2.5 was used. This lens allows the animal’s eye to be focused at a distance of approximately 2 cm, so that it occupies the entire camera field of view. This positioning enables a higher resolution in the quantification of the pupil diameter.

Low ambient illumination (white 15 lux at the rodent position) was used to avoid pupil reactions. A infrared 50 W bulb was used to illuminate the eyes. The bulb was placed at 80–100 cm in front of the rodent to get enough eye illumination, avoiding animal heating. A moisturizing eye lubricant (Lubrithal™ eye gel, Dechra Pharmaceuticals PLC, Northwich, UK) was used during the experiments to avoid corneal desiccation. An over supplement of this lubricant can cause visual artifacts, such as pupil deformation, shadows, or air bubbles that get trapped inside the gel. These artifacts must be mitigated by the experimenter, ensuring that a thin layer of lubricant is applied.

### 3.2. Control and Data-Acquisition Software

A real-time control interface was designed to monitor the pupil of both eyes and to save the video data for further processing. The control interface was developed using LabVIEW (National Instruments, Austin, TX), and was focused to solve two key aspects: temporal coherence and simplicity. The interface provides the user with the necessary controls and visual feedback from the camera images. The interface is paged in a setting tab to isolate the camera configuration and the video recording controls, ensuring a greater usability. Using this setting tab, the user can select the cameras and the gain needed to center the intensity histogram. The gain had to be adjusted at the beginning of the experiment to maximize the contrast of the acquired image in both cameras. Once the cameras were adjusted, the user selected the path to save the video recording as well as a name suffix to identify the experiment. The camera setting parameters selected for this study are shown in Table 2.

The selection of these parameters was made according to the results obtained from the functional tests performed on the prototype. In these tests, nociceptive stimuli were applied to the animal to induce responsive pupil diameter changes [24]. In the recording, a timestamp is associated to each video frame. In addition, the interface includes buttons to input actions commonly performed during a session, or to mark frames with custom label stamps.

### 3.3. Animals and Experimental Design

Experiments were performed on adult male C57BL/6J mice that were housed under standard laboratory conditions (22 °C, 12 h light/dark cycle, food and water ad libitum). Animal handling and procedures were conducted according to the guidelines of the European Commission’s directive (2010/63/EC) and Spanish law (RD 53/2013) regulating animal research. Moreover, the experimental protocols were approved by the Committee for Animal Experimentation of the University of Cadiz.

In order to monitor non-luminance-induced pupil fluctuations, the pupils of lightly isoflurane-anesthetized animals were recorded under the above-mentioned lighting conditions [24], using the CMOS camera and the micro video lens. As noxious paw compressions (PCs) elicit a robust increase in LC activity [41], pupil diameter was measured in response to repeated noxious stimulation by consecutive hind PC, applied for 1 s between the ends of a pair of surgical forceps. For each experiment, the baseline pupil size was recorded for 60 s. Subsequently, mechanical stimuli were consecutively applied four times at 100 s intervals.

Twenty mice were monitored in recording sessions to acquire images for the training and validation datasets. Total monitoring time was 8 hours 19 minutes and 21 s. A researcher marked, using a digitizing tablet, the pupil contours of a total of 1052 images. A test dataset of 636 manually annotated images was built in additional sessions with a new group of 20 animals. The rodents in this latter group were monitored, in total, for 8 hours 11 minutes and 22 s. The average time per manual frame labeling was 30 s.

### 3.4. Deep Learning-Based Method for Pupil Segmentation

Image segmentation has become a task ordinarily linked to the field of computer vision. In recent years, the advent of DL, and more specifically of convolutional neural networks (CNNs), has directly affected image segmentation, providing models with remarkable performance [42]. Image segmentation can primarily be twofold: semantic segmentation and instance segmentation [43]. While semantic image segmentation is based on the image partitioning into regions to which a certain category is assigned, instance-based segmentation allows to differentiate members of the same category through an exhaustive description of the scene [44].

Segmentation in general, and instance segmentation in particular, has benefited strongly from the adoption of CNNs to increase performance, which has led to the proposal of multiple models in very recent years [45,46,47,48,49,50,51]. This study aims at mice pupil segmentation in images acquired in a low-light environment. To this end, we opted for adapting the SOLOv2 instance-based fast segmentation framework. This novel instance-based segmentation technique was selected over semantic segmentation strategies for its outstanding performance with standard image sets, its low computational burden, and its potential to perform well while minimizing post processing [52]. Instance segmentation was revealed to be especially advantageous in managing pupil occlusions (e.g., in the case of artifacts or disproportionate brightness inside the pupil), given that the method segments the pupil as a whole, providing a single instance.

**Figure 1:**
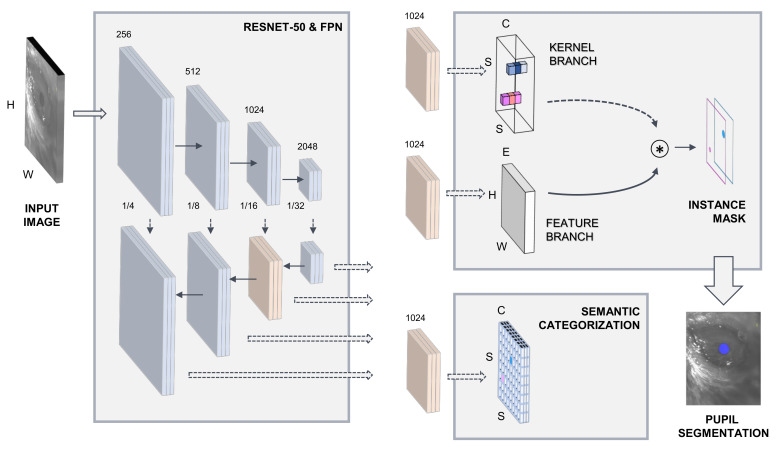
Deep learning model architecture.

As a first processing stage, SOLOv2 extracts relevant image information through a ResNet-50 convolutional backbone network [53]. SOLOv2 is pipelined with a feature pyramid network (FPN) [54]. The semantic categorization of potential image objects and instance mask extraction is carried out on two processing subnetworks. Figure 1 illustrates the model architecture. Comprehensive details on the implementation of the SOLOv2 DL-based framework can be accessed at [52].

When an experimental recording is completed by using the designed interface, an AVI file is generated. The path to this file is used as an input parameter to a script that processes the entire video using the trained DL pipeline. As output, the script provides a CSV file, including the frame number, timestamp, coordinates of the centroid of the segmented area (x,y), segmented area, pupil circularity, and distance between the farthest pixels of the region of interest (defined as the pupil diameter). Circularity was defined as:

(1) $$Circularity=4\pi \;x\;\frac{area}{perimeter^{2}}$$

The comparison of the ground truth and predicted diameters is a major outcome of this study, given that the pupillogram, defined as the curve of pupil diameter against time, is the main output system variable [55].

#### 3.4.1. Transfer Learning and Data Augmentation

A main problem encountered in the learning process of the DL algorithms consists of setting a very large number of parameters in order to generalize or learn from the train dataset. This task requires large datasets with images that feed the algorithms, but which are often limited or scarce. Generating large labeled training sets by hand is often expensive, and in many cases requires a domain expert. In this study, two techniques were used to complete the training set by increasing the number of available images: data augmentation and transfer learning.

Data augmentation was used to increase the train dataset to improve accuracy, generalization, and control overfitting. This technique allowed us to increase the size and diversity of the train dataset by generating new images from transformations of the original ones. Resizing at six different fixed scales and flipping with a probability of 0.5 were applied.

Inductive transfer learning allows pre-training a model using large labeled datasets from an unrelated problem, and then adapting it to the problem under study. This implies minor retraining, avoiding data labeling work. Transfer Learning was used in this work. All parameters of the ResNet-50 backbone network were previously pre-trained using as dataset the images provided for the Common Objects in Context (COCO) 2015 challenge [53].

#### 3.4.2. Comparison to Other State-of-the-Art Baseline Architectures

To evaluate our proposed architecture, we compared results against those of other DL-based image segmentation frameworks and against a traditional image processing pipeline not rooted in machine learning techniques. State-of-the-art methods based on DL, belonging to both the fields of instance and semantic segmentation, were chosen as reference algorithms for comparison. The selected semantic segmentation architectures were based on DeepLabv3+, which uses atrous convolutions and spatial pyramid pooling [56], and SegNet [57]. The DeepLabv3+ architecture was implemented considering four different backbone networks, namely: Xception [58], ResNet-50, and ResNet-18 [53], and MobileNetV2 [59]. To compare with a state-of-the-art architecture in instance segmentation, we implemented the Mask R-CNN architecture, a top-down approach of segmentation-by-detection that includes a ResNet-50 backbone [45]. Finally, an additional baseline experiment was conducted. To this purpose, a conventional image processing algorithm, following common operations performed in state-of-the-arts LC studies was applied. This segmentation algorithm included the manual selection, in the first video frame, of a bounding box around the pupil area. The pupil detected inside this box served as an initial reference for its location and its mean gray level (pixel intensity). In successive frames, a thresholding segmentation was applied on the image using as threshold the average intensity value of 13 pixels located inside the pupil detected just in the previous frame. As a result, an image with different areas or regions of connected pixels was obtained. In a post-processing step, small regions and those outside the initial bounding box were removed. Finally, the pupil was segmented as that region whose centroid was closest to the pupil centroid detected in the previous frame.

For a fair comparison, we retrained the DL-based image segmentation networks on our datasets.

### 3.5. Validation, Testing, and Evaluation Metrics

**Figure 2:**
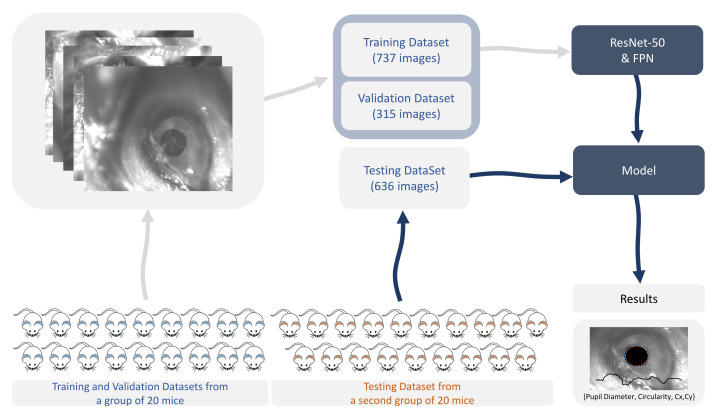
Training, validation, and testing strategy. FPN: feature pyramid network. Cx,Cy: coordinates of the centroid of the segmented area.

A total of 1688 images were used for training, validation, and testing. The training set included 737 images, the validation dataset had 315 images used for validation during training, and the test dataset included 636 images. The training, validation, and testing adopted strategy is depicted in Figure 2.

Among the performance metrics broadly used in recently published segmentation studies, Intersection over Union (IoU), mean Intersection over Union (mIoU), pixel accuracy, and mean accuracy were selected as candidates for this work. While Intersection over Union (IoU) estimates the segmentation performance by calculating the intersection and union between the ground truth and the prediction, mIoU takes the IoU over all of the classes and averages them. Formally, these metrics are expressed as:

(2) $$Pixel\;Accuracy=\frac{\sum_{i}p_{ii}}{\sum_{i}t_{i}}\;,\;\;Mean\;Accuracy=\frac{1}{k}\sum_{i}\frac{p_{ii}}{t_{i}}$$

(3) $$mIoU=\frac{1}{k}\sum_{i}\frac{p_{ii}}{t_{i}+\sum_{j}p_{ji}-p_{ii}}\;,\;\;IoU=\frac{p_{ii}}{\sum_{j}p_{ij}+\sum_{j}p_{ji}-p_{ii}}$$

where *k* is the number of classes in ground truth segmentation (k=2 in this study), $p_{ij}$ is the number of pixels that actually belong to class *i* and that have been classified as belonging to class *j*, and $t_{i}$ is the total number of pixels of class *i* found in the ground truth segmentation [60]. The values of mIoU and IoU are restricted to [0,1] interval, with 1 representing 100% accuracy, and 0 corresponding to 0% accuracy.

The main disadvantage of using pixel accuracy is that the result might look good if one class overpowers the other. Indeed, this situation is encountered in the problem of pupil detection from a whole image of the animal’s eye. The region of interest is small compared to the image background. This also makes mIoU an ill-suited measure. However, IoU applies an averaging across classes. Consequently, the model performance was verified by calculating the IoU for the entire dataset [61].

In addition to IoU, the mean absolute percentage errors (MAPE) of the pupil diameter, circularity, and centroid coordinates were calculated. MAPE, a popular metric commonly used to evaluate prediction performance, is given by:

(4) $$MAPE=\frac{1}{N}\sum_{t}|\frac{G_{t}-P_{t}}{Gt}|$$

where $G_{t}$ and $P_{t}$ represent, respectively, the actual (ground truth) and predicted pupil diameters in frame *t*, and N is the number of evaluated images.

### 3.6. System Configuration and Training Details

Training, validation, and testing of the segmentation model was accelerated with the support of a NVIDIA^®^ DGX station. An initial learning rate, weight decay, and momentum of 0.001, 0.0001, and 0.8 were, respectively, selected. Hyperparameter tuning was not applied in this study. The loss function used for training was the cross-entropy function for semantic pixel-wise segmentation architectures. In the case of instance segmentation methods, the training loss function was defined as follows:

$$L=L_{cate}+\lambda L_{mask}$$

where $L_{cate}$ is the conventional Focal Loss [62] for semantic category classification and $L_{mask}$ is the dice loss for mask prediction [63]. Input images to the segmentation model were previously scaled to 640×512 pixels.

### 3.7. Statistical Analysis

In addition to estimating the performance metrics defined in the previous section for both the proposed and baseline methods, a number of statistics was calculated for the proposed SOLOv2-based approach. Concordance between measurements of pupil diameter was assessed by correlation coefficient, regression analysis, scatter plots, and the limits of agreement (LoA) on the Bland–Altman plot [64]. A probability of error <5% was considered significant. The Bland–Altman method is a graphical method that describes the agreement between two quantitative measurements quantifying the mean difference between them (bias) and a confidence range, which is expected to include 95% of the differences between two different measurement techniques.

## 4. Results and Discussion

### 4.1. Mechanical Framework, Hardware, and User Interface

**Figure 3:**
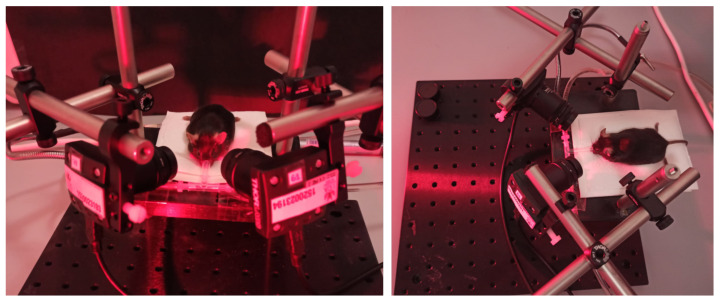
A close-up of the experimental setup during a pupil recording session.

The developed platform and user interface described in Section 3 were used to record pupil images from 40 mice. Figure 3 illustrates the experimental setup during one of the experiments developed in the laboratory environment from two viewing perspectives. The picture shows the positioning of the two video cameras and the animal’s resting platform. The rodent receives the anesthesia through a nasal mask. The light’s reddish hue is due to infrared illumination.

### 4.2. Control and Data-Acquisition Software

**Figure 4:**
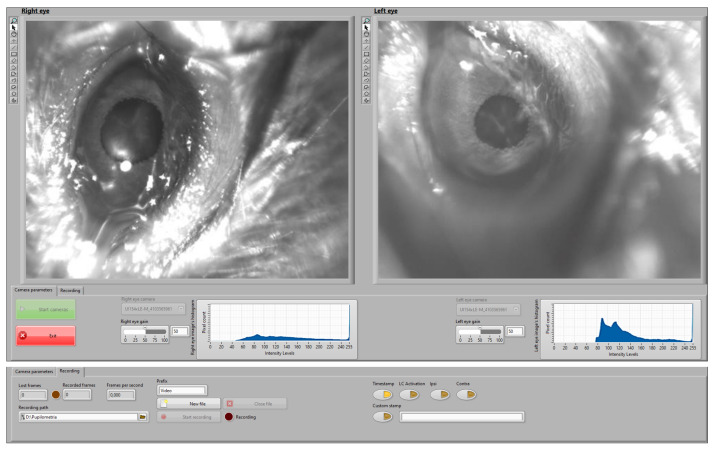
User interface developed to guide the monitoring and recording process. Top part: video settings page. Bottom part: camera settings page.

The real-time control interface allows monitoring the pupil and recording video data during the experiments. Figure 4 illustrates the implemented control interface during a recording session. The camera setting page (bottom part of Figure 4) allows defining the setting of the cameras while live-checking the intensity histogram. The video setting page (top part of Figure 4) enables the user to access the recording controls, defining the path for the recorded file and checking lost and recorded frames as well as the frame ratio. The user can use the programmed buttons to include video stamps with predefined or personalized labels. The interface is prepared to integrate other signals or to synchronously trigger stimuli (e.g., optogenetic stimulation).

### 4.3. Segmentation Assessment

We compared the proposed architecture to other baselines. For a fair comparison, we trained the image segmentation networks detailed in Section 3.4.2 on our datasets. The segmentation metrics considered in this study were calculated for the validation and testing of the pupil segmentation models. MAPE of the predicted pupil size, circularity, and centroid coordinates, as well as the IoU metrics are presented for both datasets. The IoU was determined to compare the ground truth and predicted pupil masks. A detailed analysis of the results is given in Table 3.

Results show that our architecture achieves a superior segmentation performance (higher IoU coefficient) with respect to the evaluated off-the-shelf instance (Mask R-CNN), semantic (DeepLabv3+ and SegNet) and traditional segmentation models. These results point out that pupil segmentation under the described experimental setting remains a challenge.

The mean IoU values achieved by the proposed architecture on the validation and test sets were 0.94 and 0.93, respectively. These IoU values outperformed those of the rest of the methods. Only the Mask R-CNN instance segmentation method equaled this metric in the test set (0.93).

The MAPE error for the pupil diameter estimated for our SOLO-2-based approach was 1.70% in the validation set, and 1.89% in the test set. This error is substantially lower than the errors measured with the other reference methods. An analogous situation occurs with the pupil circularity measurement. In both cases, the methods based on instance segmentation provided better performance than the others. The proposed method was slightly outperformed in accuracy by the Mask R-CNN architecture in calculating the coordinates of the pupil center point.

The purpose of this study is to provide a system for accurate quantification of the pupil diameter rather than pupil position, which is more useful in studies focused on gaze tracking. To this end, the proposed algorithm outperforms the reference methods in our datasets. In addition, the mean pupil detection time using the trained SOLOv2-based model, once the input image was scaled, was 0.03 s on average, while the mean processing time using the Mask R-CNN model was 0.13 s. This makes the proposed method a more effective approach from a computational perspective.

**Figure 5:**
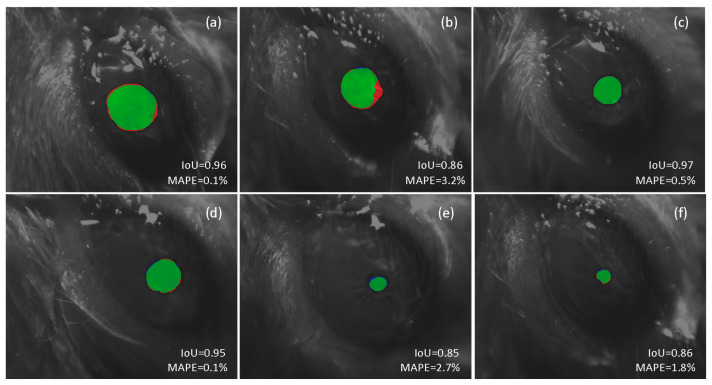
Comparison between automatic and manual pupil segmentation. The red color shows the result of the manual segmentation (ground truth) and the blue color shows the result of the proposed deep learning-based architecture. The intersection of both regions is shown in the green color. The Intersection over union (IoU) for the predicted segmented region and mean absolute percentage error (MAPE) for the pupil diameter estimation are shown in each case.

Figure 5 illustrates the result of using our approach for automatic segmentation for a set of six frames, ordered from largest (a) to smallest (d) pupil size, captured under different conditions, some of them considered particularly challenging to process automatically. Figure 5a,b show the results obtained in two images of dilated pupils. The presence of vibrissae and moisturizing gel in addition to a recording viewing perspective that accentuates the sphericity of the ocular globe make automatic segmentation using conventional techniques particularly troublesome. However, the proposed DL-based framework operates fairly well in both cases, showing a high IoU and a small mean absolute percentage error (MAPE) when estimating the pupil size. The processing of contracted pupil images is equally challenging. In these cases, a little deviation in the contour estimate translates into a larger percentage error. In addition, we may also take into account potential bias during the annotation process by the human experimenter in such small regions for what it is likely to deviate some pixels from the perfect segmentation. This situation is consistent with the findings in [55]. Figure 5e illustrates an example of such a scenario. The differences in the automatically segmented region, with respect to the ground truth segmentation, leads to a greater error in the pupil size estimate.

**Figure 6:**
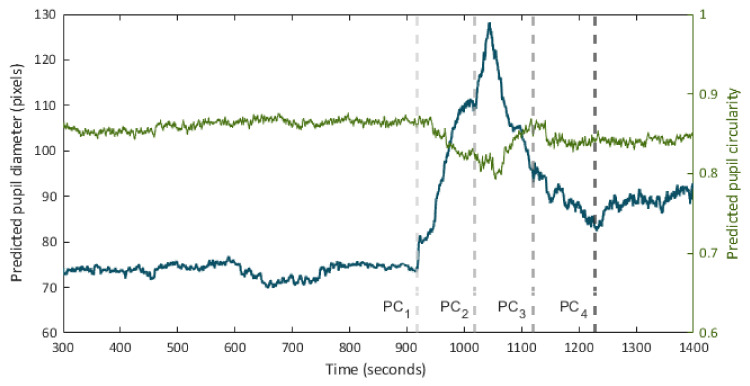
Time series of pupil diameter (pupillogram) and circularity over the course of a pupil recording session. Times $t=PC_{i}$ point out the onset of consecutive hind paw compression (PC) events.

Figure 6 presents the predicted pupillogram and the circularity diagram in one of the pupil recording experiments.

**Figure 7:**
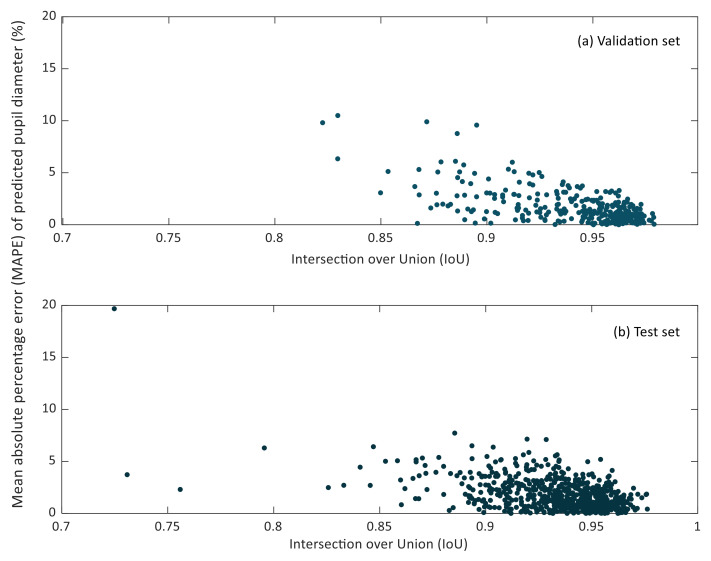
Scatter plot of the intersection over union (IoU) versus the mean relative percentage error (MAPE) of predicted pupil diameters in the validation (**a**) and test (**b**) sets.

**Figure 8:**
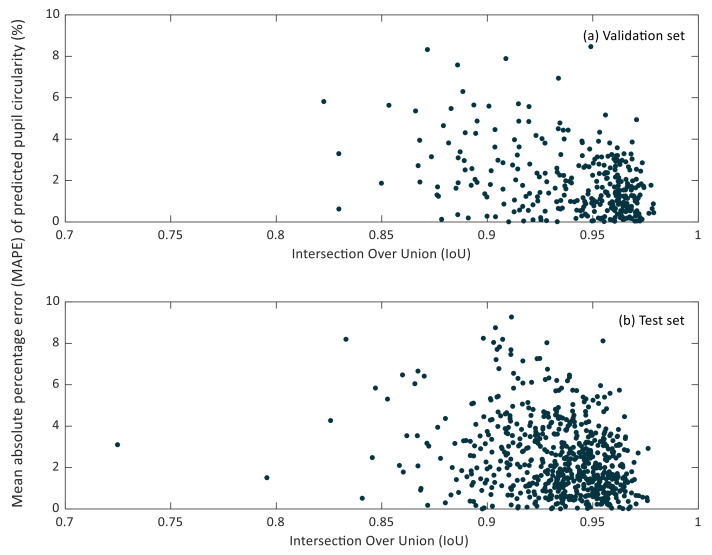
Scatter plot of the intersection over Union (IoU) versus the mean relative percentage error (MAPE) of predicted pupil circularity in the validation (**a**) and test (**b**) sets.

In Figure 7 and Figure 8, the relationship between the IoU and MAPE of pupil diameter and pupil circularity in the validation and test sets can be observed.

It can be noticed that small IoU values do not lead to considerable MAPE values of the predicted pupil diameter. Lower IoU values correspond to cases in which the pupil region has been segmented partially. However, it may happen that the missing region in the resulting segmentation does not affect the estimation of the so-defined pupil diameter, as can be recognized in Figure 5b.

It can be appreciated how the proposed method is effective for the generation of the pupillogram in long-duration experiments, enabling the detection of the changes in the pupil diameter in response to the different stimuli presented to the animal. The system also allows the recording of the observed changes in the circularity of the detected region. In addition, the processing time per video frame is lower than the frame rate during recording, which allows to display the segmentation results in real-time, allowing the experimenter to know, in situ, the results by synchronously observing changes in the pupil diameter and circularity under different experimental conditions.

### 4.4. Statistical Analysis

This subsection includes a more in-depth statistical analysis of the diameter and circularity data provided by the proposed method, in order to corroborate the validity of the measurements provided and to delimit the LoA.

Normal distribution of data (pupil diameter and circularity) was evaluated using the D’Agostino and Pearson test and the Kolmogorov–Smirnov test. No normality tests were passed and consequently, non-parametric rank correlation coefficients (Spearman’s rho and Kendall’s tau) were estimated.

Spearman and Kendall coefficients of rank correlation showed a strong positive association between the values obtained manually and by the proposed automatic framework. The result demonstrated excellent correlation between the methodologies: ρ = 0.997, τ = 0.951, *p*-value < 0.0001.

**Figure 9:**
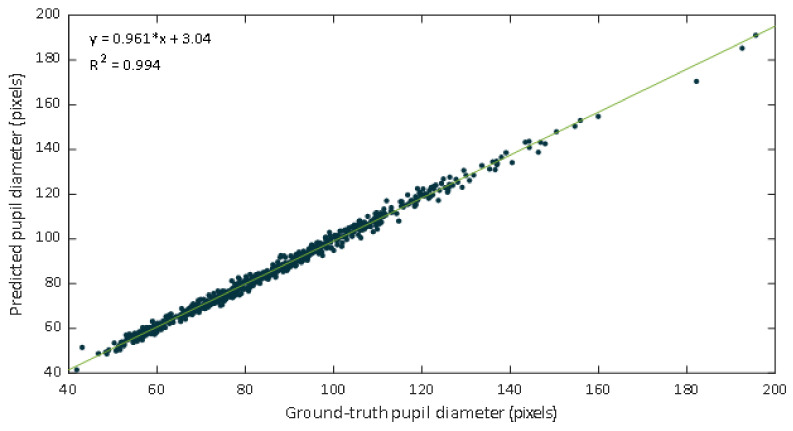
Scatter plot of the ground truth pupil diameter versus the estimates of our proposed approach.

Data were inspected through the representation of a scatter diagram. The regression lines and the coefficients of determination were calculated. Figure 9 shows a scatter plot of the manual labeled pupil diameters versus values estimated from the DL-based approach. The fitted line was y=0.961x+3.04, the $R^{2}$ value was 0.994, and the Pearson correlation coefficient was 0.997.

**Figure 10:**
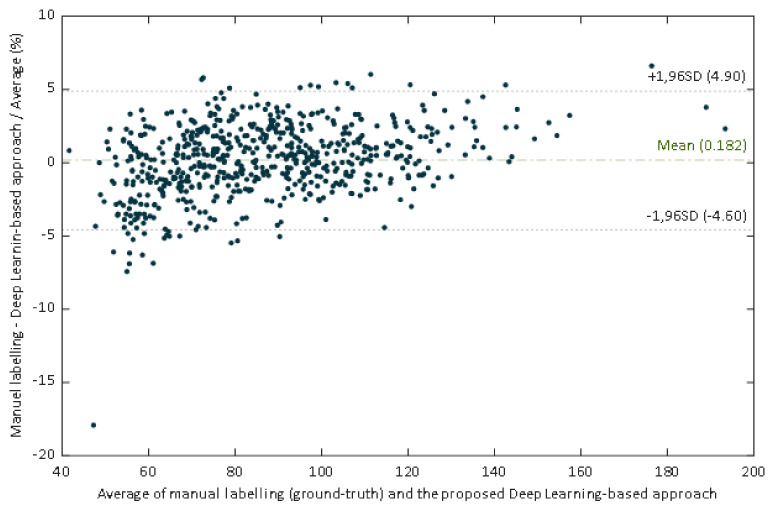
Scatter plot of the ground truth pupil diameter versus the estimates of our proposed deep learning-based approach.

The regression line have a slope of 0.961, which could be evaluated as a very good agreement between methods. In this case the slope indicates that the proposed DL-based approach slightly underestimates the manual estimation. However, a high correlation is not necessarily synonymous with agreement between methods, since it evaluates the relationship and not the difference. Since Bland Altman analysis may overcome this limitation, this methodology was also applied. The performed Bland–Altman concordance analysis demonstrated relatively small dispersion for the manual procedure compared with the DL-approach (Figure 10). The Bland–Altman plot depicts an excellent agreement. The relative bias was 0.182% being the 95% confidence interval (CI) of (–0.007%, –0.371%). The lower and upper 95% LoA were (–4.90%, –4.26%), and (4.62%, 5.27%), respectively.

The obtained statistical measures confirm that the performance of the proposed framework is suitable for real-time pupil size tracking. The system has demonstrated excellent robustness in mice with low pupil–iris contrast. Published studies describing systems with a similar purpose scarcely present the obtained performance by reporting common accepted segmentation metrics. As an exception, and very recently, an average relative diameter error of 12% and a median relative error of 4% has been reported for a machine learning algorithm applied to rat pupil images [55].

Additionally, a major advantage of our approach is the lack of need for any manual adjustment. In [6,21,30,31], the user is required to make, at some point, or even repeatedly throughout the process, manual adjustments to the parameters of the detection algorithms. In contrast, the method proposed in this study can operate fully automatically.

## 5. Conclusions

In this work, a pupillometry system, including a novel DL-based tool to detect and measure real-time changes in pupil size in lightly anesthetized mice, was described, discussed, and evaluated. The system performance was assessed using an external test set, resulting in an IoU of 0.93 and a MAPE of the predicted pupil diameter of 1.89%. These results demonstrate the ability of the proposed architecture to outperform other state-of-the-art reference methods, whether based on instance segmentation, such as Mask R-CNN, semantic segmentation, such as SegNet and DeepLabv3+ with different backbones (Xception, ResNet-50, ResNet-18, and MobileNetV2), or classical image processing techniques. In addition, the Bland–Altman analysis showed a great level of agreement. Furthermore, the resulting processing time per frame was lower than the frame rate, which ensures the real-time system capability.

The evaluated segmentation model showed good performance regarding widely used similarity segmentation metrics and success rates. This is of particular relevance given that the datasets included pupil images with high variability in sizes, light intensity, and eye artifacts. In this regard, preprocessing stages were not performed to grayscale color images before the segmentation process, meaning that the trained DL-based segmentation model appears to be consistent to non-uniformities that can arise among images, such as intensity variations or distance to the animal during the recording process. These results indicate that both the combined use of the designed mechanical setup, the user interface, and the method based on artificial intelligence and supported by DL, enable the detection of spontaneously and elicited pupillary changes and, therefore, can be used to monitor the mouse’s LC activity state during experiments with lightly anesthetized animals.

This work has limitations. System robustness could potentially be improved by adding preprocessing steps or enriching the training set so that the system can manage events, such as vibrissae, unfocused areas, or light reflection. The search for optimal hyperparameters for the proposed DL network remains to be explored for its potential to improve results. In addition, images used in this research were acquired under the same experimental conditions, by the same experimenter, and using the same acquisition parameters. Augmenting the training set with additional images from other sources could contribute to achieve an improved results generalization.

In conclusion, the outcomes of the proposed hardware and software tools reveal that the presented strategy provides direct instance segmentation and offers state-of-art results with a lower computational burden, independently from the pupil’s size, light intensity, or any other features typical of the recording process in anesthetized mice. Although this study was performed to assess indirectly the function of the LC-noradrenergic system in anesthetized rodents, the presented framework could be used as an accurate analysis tool in any neurophysiological study in which the animal is lightly anesthetized or has its head fixed.

## 6. Patents

The mechanical framework presented hereby is currently patent pending.

## Figures and Tables

**Table 1:** Studies recently conducted to evaluate the function of the locus coeruleus using pupillometry recordings.

Authors	Year	Vision Hardware	Light Source	Binocular System	Method to Estimate the Pupil Size
Hayat et al. [31]	2020	Color cameras	Infrared light	No	Image analysis pipeline using MATLAB
Privitera et al. [21]	2020	Raspberry Pi 3 Night vision camera	Infrared light	No	Image analysis pipeline using MATLAB and, alternatively, a deep neural network (DeepLabCut)
Zerbi et al. [23]	2019	Raspberry Pi 3 Night vision camera	White and infrared light	No	Image analysis pipeline using MATLAB
Breton-Provencher and Sur [30]	2019	High-resolution CMOS camera 1.0× telecentric lens	Infrared light	No	Image analysis pipeline using MATLAB
Yüzgeç et al. [32]	2018	0.3 MP USB cameras Micro-video lens 25.0 mm, f/2.5	Infrared-back illumination	Yes	Image analysis pipeline using MATLAB
Liu et al. [24]	2017	Pupillometry system assembled in-house	White light	Yes	Not detailed
Reimer et al. [6]	2016	High-resolution CMOS camera 1.0× telecentric lens	Red and green light	No	Image analysis pipeline using LabVIEW and MATLAB

**Table 2:** Setup parameters used for image acquisition during the experimental sessions.

Setup Parameter	Value
Pixel Clock	30 MHz
Frame rate	10 fps
Exposure time	79.085 ms
Image size	1280 × 1024 px
Format	Mono 8 bits per pixel
Gain	User adjustable

**Table 3:** Comparison of prediction performance of the proposed architecture and baselines in the validation and test sets. IoU: Intersection over Union. MAPE PD: mean absolute percentage error of pupil diameter. MAPE PC: mean absolute percentage error of pupil circularity. MAPE Cx: mean absolute percentage error of X-Centroid coordinate. MAPE Cy: mean absolute percentage error of Y-Centroid coordinate.

	Validation Set/Test Set				
Method	IoU	MAPE PD (%)	MAPE PC (%)	MAPE Cx (%)	MAPE Cy (%)
Mask R-CNN	0.92/**0.93**	4.13/2.98	2.11/3.28	0.30/**0.18**	**0.30**/**0.23**
DeepLabv3+ ResNet-50	0.90/0.86	4.25/5.28	2.72/4.45	0.45/0.73	0.57/0.91
DeepLabV3+ ResNet-18	0.84/0.79	7.80/7.94	11.95/13.19	0.86/1.05	0.82/1.14
DeepLabv3+ MobileNetV2	0.87/0.87	5.87/5.88	7.28/4.94	0.70/0.51	0.62/0.52
DeepLabV3+ Xception	0.80/0.77	11.71/10.06	11.14/9.41	1.30/0.94	1.19/1.36
SegNet	0.74/0.70	16.85/22.24	80.15/76.62	1.77/0.76	1.49/0.72
Traditional Algorithm	0.70/0.80	13.43/8.38	24.55/10.46	2.18/1.50	2.42/1.05
Proposed method	**0.94**/**0.93**	**1.70**/**1.89**	**1.89**/**2.56**	**0.26**/0.26	0.36/0.38
